# Evaluation of Pharmacokinetic Drug–Drug Interactions: A Review of the Mechanisms, In Vitro and In Silico Approaches

**DOI:** 10.3390/metabo11020075

**Published:** 2021-01-27

**Authors:** Yaru Peng, Zeneng Cheng, Feifan Xie

**Affiliations:** Division of Biopharmaceutics and Pharmacokinetics, Xiangya School of Pharmaceutical Sciences, Central South University, Changsha 410013, China; yaru.peng@hotmail.com (Y.P.); chengzn@csu.edu.cn (Z.C.)

**Keywords:** drug–drug interactions, pharmacokinetics, physiologically based pharmacokinetic model, cytochrome P450, transporter

## Abstract

Pharmacokinetic drug–drug interactions (DDIs) occur when a drug alters the absorption, transport, distribution, metabolism or excretion of a co-administered agent. The occurrence of pharmacokinetic DDIs may result in the increase or the decrease of drug concentrations, which can significantly affect the drug efficacy and safety in patients. Enzyme-mediated DDIs are of primary concern, while the transporter-mediated DDIs are less understood but also important. In this review, we presented an overview of the different mechanisms leading to DDIs, the in vitro experimental tools for capturing the factors affecting DDIs, and in silico methods for quantitative predictions of DDIs. We also emphasized the power and strategy of physiologically based pharmacokinetic (PBPK) models for the assessment of DDIs, which can integrate relevant in vitro data to simulate potential drug interaction in vivo. Lastly, we pointed out the future directions and challenges for the evaluation of pharmacokinetic DDIs.

## 1. Introduction

Drug–drug interactions (DDIs) can occur in patients undergoing polytherapy at pharmacokinetic (PK) and pharmacodynamic (PD) level, resulting in altered drug concentrations by either inhibiting or inducing the enzymes or transporters responsible for the disposition of that drug or producing agonistic or antagonistic effects [1]. Both the PK and PD interactions may lead to reduced efficacy or increased toxicity in the clinic. Compared to PD DDIs, which need a case-by-case evaluation [2,3], PK DDIs are more common and have received great attention from drug agencies [4,5]. Early termination of development, refusal of regulatory approval, and market withdrawals due to PK DDIs have been widely reported [6,7]. For example, in 1997, the US Food and Drug Administration (FDA) recalled the first non-sedating antihistamine-terfenadine due to its potential to reach lethal blood exposing levels when co-administered with some antibiotics like erythromycin and ketoconazole [8].

To mitigate the risk of costly developmental failure or undesired therapeutic outcomes, evaluation of DDIs has been integrated into the drug discovery stage and post-marketing administration. Traditionally, if in vitro studies indicate a high-risk for PK-related DDIs, a mechanistic static model will be applied, estimating the ratios of concentration area under the curve (AUCi/AUC) for cytochromes P450 (CYPs)- and transporter-based inhibition or induction in the presence (AUCi) or absence (AUC) of the perpetrator. However, most static models tend to overpredict the extent of DDIs since they are usually based on the maximum concentration of perpetrating drugs. Moreover, the static model approach assumes the same precipitant concentration, CYPs, and transporter expression levels across the population; thus, it only provides average estimates of the DDIs and the risk to individuals is not evaluated [9].

In recent years, physiologically based pharmacokinetic (PBPK) modeling is widely adopted by the pharmaceutical industry for DDIs evaluation because of its superior power to bridge in vitro and in vivo DDIs studies and the ability to predict complex DDIs in patient populations with various administration schemes [10,11,12,13]. As a mechanistic dynamic model, the PBPK approach is highly appreciated by the agencies for new drug applications and is gradually replacing the empirical static methods. CYPs hold a central position for DDIs studies since they account for the metabolism of about 45% of the marketed drugs [14]. Currently, the in vitro assays and in vitro-in vivo extrapolation (IVIVE) methods for CYPs meditated DDIs are quite mature and extensively reported [15,16,17]. Besides the CYPs-DDIs, the DDIs are caused by non-CYP enzymes and transporters, such as UDP-glucuronosyltransferases (UGTs), uptake transporters (OATPs, OATs, and OCTs) and efflux transporters (*P-gp*, BCRP), are also important but are poorly studied and less understood [18,19]. The power of PBPK modeling for transporters mediated DDIs is obvious, and some excellent cases for the OATPs transporter have been reported [20,21,22], and some new sights have been emerged for *P-gp* and BCRP recently [23,24,25]. To our knowledge, the static and dynamic models towards CYPs-DDIs are well reviewed [1,9,18,26], while a comprehensive summary of the static and dynamic models for both CYPs- and transporters- DDIs are scarce.

In this review, we provide an overview of the in vitro tools, conventional static approaches, and dynamic models for PK DDIs, where the CYPs and transporter-meditated inhibition or induction are covered. In addition, the limitations of these methodologies and future perspectives for handling PK-related DDIs are discussed.

## 2. Mechanisms for CYPs/Transporter-Mediated PK DDIs

As stated by the health authorities, CYPs are of high clinical relevance for DDIs. The CYPs constitute the major enzyme family capable of catalyzing oxidative biotransformation. Of the 57 putatively functional human CYPs, only about a dozen enzymes, belonging to the CYP 1, 2, and 3 families, are responsible for the biotransformation of most xenobiotics, including 70–80% of all drugs in clinical use [6]. CYPs 3A4, 2C9, 2C8, 2E1, and 1A2 are the highest expressed forms in the liver, while 2A6, 2D6, 2B6, 2C19, and 3A5 are less abundant, and CYPs 2 J2, 1A1, and 1B1 are mainly expressed extrahepatically [27]. The important CYP isoforms for DDIs are 1A2, 3A4/5, 2B6, 2C8, 2C9, 2C19, and 2D6 [4,28], and CYP3A4/5 is of the highest interaction ratio [2]. The high permeability drugs, mainly eliminated by metabolism, are often involved in CYPs-mediated DDIs.

**Figure 1:**
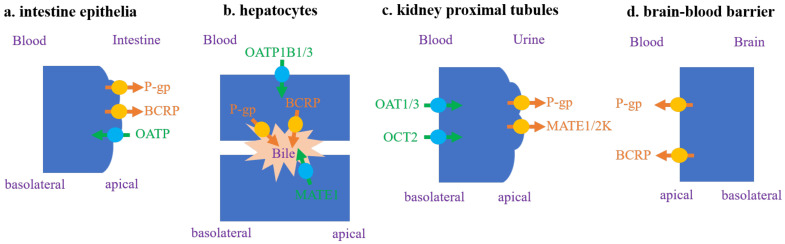
Transporters with a high clinical interest in drug–drug interactions (DDI) evaluation [4,28].

Drug transporters are also crucial for DDIs. They are expressed in a variety of organs, including the intestine, liver, kidney, and brain, and they play a key role in the in vivo drug disposition, adverse reactions, and therapeutic efficacy of drugs [29]. The well-known transporters involved in DDIs are *P-gp*, BCRP, OATP1B1/OATP1B3, OAT1/OAT3, OCT2 and MATE1/MATE2K (Figure 1), and the poorly permeable drugs, predominantly eliminated by renal and/or biliary excretion, often suffer from transporter-mediated DDIs [30]. The hepatic uptake transporters are located on the basolateral membrane and mediate uptake or bidirectional transport of substrate drugs, and hepatic apical efflux transporters could take the drugs out of the hepatocytes into the blood or bile compartments. Inhibitions of hepatic uptake transporters, such as OATP1B1/3, play a significant role in transporter-mediated DDIs, mediating more than half of the severe DDIs with AUC changes > 5-fold [2,31]. Among the efflux transporters, interactions with *P-gp* are the most studied [32,33]. Due to the overlapping substrate specificity of *P-gp* and CYP3A, an inhibition of *P-gp* in the gut and liver can impact exposures of CYP3A substrates. Inhibition of *P-gp* may not result in clinically significant differences in the plasma exposure of the victim drug, but it can attenuate the efficacy of drugs targeting barrier tissues such as the brain, lymphocytes, and tumor [34].

A candidate drug should be evaluated for its potential to be a perpetrator (inhibits or induces enzymes or transporters) or victim (whose PK is changed by a perpetrator) of DDIs with specific probe substrates, selective inhibitors or inducers, and second, with likely co-administered drugs. The alteration of drug-metabolizing enzyme activities can occur via three primary mechanisms: (1) reversible inhibition, (2) mechanism-based inactivation (MBI), and (3) induction [35]. The reversible mechanisms of inhibition can be competitive, noncompetitive and uncompetitive. Competitive inhibition, occurring in most cases, involves the inhibitor competing directly with the substrate for binding to the active site of the enzyme. In noncompetitive inhibition, the inhibitor binds at the allosteric site independently of substrate binding, meaning the inhibitor shares the same affinity for both enzyme and enzyme–substrate complex. This activity differentiates noncompetitive inhibition from uncompetitive inhibition, in which an inhibitor binds only to the enzyme–substrate complex [36]. MBI occurs when a compound undergoes a catalytic transformation by an enzyme to a species that, prior to release from the active site, inactivates the enzyme by either covalent or non-covalent binding. This indicates that the inhibitory effect may persist in vivo even after elimination of the inactivating species, and that the active enzyme can only be recovered by de novo synthesis. Induction occurs when a compound upregulates the synthesis of enzyme via receptor-mediated mechanisms (aryl hydrocarbon receptor (AhR) for CYP1A, constitutive androstane receptor (CAR) for CYP2B6, and pregnane X receptor (PXR) for CYP3A), affecting the transcription, translation and expression of that protein yielding a higher abundance within a given tissue mass [37].

Transporters-mediated DDIs follow the same inhibition or induction principles of CYPs-mediated DDIs [18,38]. The inhibition mechanism for most transporters is usually considered as only competitive, while OATP1B1/3 has been found to be inhibited by a few inhibitors via MBI [39,40,41]. The induction of transporters is most reported for *P-gp* [2].

## 3. In Vitro Tools for Determining Key PK DDIs Parameters

The evaluation of DDIs generally involves multiple processes, including mechanism clarification from preclinical/in vitro systems, model-based predictions, and final confirmation with clinical studies [42]. Due to the species difference in metabolizing enzymes and transporters between humans and animals, preclinical animal DDIs studies are not frequently adopted. Currently, the evaluation of DDIs usually begins with the in vitro experiments to determine the interaction mechanism and critical interaction parameters for the perpetrators and victims, and the derived parameters could be further implemented to the static and dynamic models.

### 3.1. Inhibition or Induction Potential of a Perpetrator

Preclinical prediction of CYPs inhibition mediated DDIs have been performed conventionally using the well-characterized and intensively studied human liver microsomes (HLM) and human hepatocytes (hHEPs) [42].

For reversible CYPs or transporter-mediated inhibition, the rate of turnover of the probe substrate is determined in the absence or presence of an inhibitor, using a matrix of varying substrate and inhibitor concentrations. Non-linear regression analysis is applied to the data to assess the type of inhibition and to obtain an estimate of the absolute inhibition constant (*K_i_*). For a competitive enzyme inhibition, *K_i_* is given by Equation (1) based on the Cheng–Prusoff equation [43], where *IC_50_* is the inhibitor concentration required to inhibit 50% of the metabolic rate of a probe substrate, *S* is the substrate concentration, and *k_m_* is the Michaelis–Menten constant for the substrate. Regarding the inhibition of transporters, the *IC_50_* was often used as a practical substitute for the *K_i_*, and the common in vitro systems for quantifying the inhibitory potency of transporter inhibitors are Caco-2 cells, HEK or MDCK transfected cells for uptake transporters, and membrane vesicles for efflux transporters [28].

(1) $$K_{i}=\frac{IC_{50}}{1+\frac{S}{K_{m}}},$$

Estimation of the inactivation parameters of MBI integrates the steps of pre-incubation with different inhibitor concentrations for varying times, quenching with a dilution of the reaction mixture and further incubation after addition of the probe substrate in HLMs or hHEPs in plasma, followed by simultaneous fitting of the combined data set to obtain estimates of all in vitro kinetic parameters [1]. The pseudo-first-order rate constant (*k_obs_*) is related to the maximum inactivation rate (*k_inact_*) and the concentration required for half-maximal inactivation (*K_I_*) as Equation (2) (equally applies to transporter involved MBI). The CYP natural degradation rate constant (*k_deg_*) for the inactivated isoform is another key parameter for MBI. However, due to the challenges with methodology and sample size, there is a lack of consensus on turnover half-life (*t_1/2_*) and a paucity of reported *k_deg_* values for many of the CYPs involved in clinically relevant DDIs [44]. For hepatic CYP3A4, *k_deg_* ranges from 0.0077 to 0.03 h^−1^ [45]. These values have been measured by three main in vitro approaches (1) measuring CYP apoprotein expression loss in liver models over time [46], (2) induction of CYP enzymes followed by tracking of de-induction recovery profiles [47], and (3) pulse-chase analysis after de-induction [48].

(2) $$k_{obs}=\frac{k_{inact}\times I}{K_{I}+I},$$

The most relevant in vitro models for studying drug induction are those using freshly cultured or cryopreserved hepatocytes [37]. The induction endpoint can be assessed via enzyme activities (most common), messenger RNA (mRNA) and CYPs protein content measurement (mechanistic). The magnitude of the induction effect can be described according to a calibration curve of relative induction scores (*RIS*), which is given by Equation (3). *E_max_* is the maximum induction capacity, and *EC_50_* is the effective concentration of the inducer at half-maximal induction. Due to the similarities of induction mechanism between CYPs and transporters, the induction equation for characterizing CYPs induction studies can also be applied to transporters induction studies, but currently, the in vitro systems to evaluate the induction of *P-gp* and other transporters are not well established [28].

(3) $$RIS=\frac{\;E_{max}\times I_{max,\mu }}{EC_{50}+I_{max,\mu }},$$

### 3.2. Reaction Phenotyping for Victim

For an accurate DDI prediction, it is important to quantitatively characterize relative contributions of the major enzyme(s) and/or transporter(s) involved in the clearance of victims, which is indicated by the parameters of fraction metabolized (*f_m_*) and fraction transported (*f_t_*). If an *f_m_* or *f_t_* value is greater than 0.25, the interaction between a victim drug and inhibitors/inducers of the relevant enzyme/transporter is considered significant [28].

The determination of *f_m_* requires the identification of specific enzymes involved in a drug’s metabolism. In vitro reaction phenotyping typically involves the incubation in HLMs or hHEPs with chemical inhibitors or antibodies and human recombinant P450 enzymes(rh-CYPs) [49,50]. By performing a series of incubations with various selective inhibitors for each of the main CYPs in HLMs or hHEPs, and comparing the relative metabolism rates (determined by measuring parent depletion or detecting metabolite formation), one can identify which inhibitor reduces the overall metabolism to the greatest extent and thereby uncover the metabolic pathway that contributes the most to the clearance of a compound. The percentage of inhibition observed will be the *f_m_* for each enzyme [50,51]. To quantitatively translate the rh-CYPs data to *f_m_* in HLMs or hHEPs, certain correlation factors are required, such as inter-system extrapolation factor (ISEF) or relative activity factor (RAF) [50].

Regarding the estimation of *f_t_*, similar approaches based on RAF or relative expression factor (REF) have been proposed. For example, a hepatic clearance drug with a rate-determining process of the hepatic uptake across the basolateral membrane [52,53], the RAF and REF methods can be used to determine the *f_t_* of the uptake transporters such as OATP1B1/3 [54,55]. The RAF method estimates the differences between the uptake rate in hHEPs (expresses multiple uptake transporters) and transfected cell lines with a specific transporter using relatively selective substrates, such as pitavastatin [54]. REF is usually obtained via Western blot analysis or LC–MS/MS-based protein quantification. Additionally, phenotyping approaches based on selective inhibitors or gene knockdown in primary hepatocytes can be employed to assess *f_t_* [56,57]. The value of *f_t_* is estimated by the uptake clearance in the absence (control) and presence of inhibitors, formulated as *f_t_* = 1 − (uptake clearance with each selective inhibitor/uptake clearance of control). These aforementioned strategies can also be applied for estimating the contribution of other transporters, such as the OATs/OCTs involved in renal tubular secretion.

## 4. Conventional Static Models for PK DDIs

### 4.1. Basic Model

PK DDIs is often evaluated by the overall change in the exposure of victim drug according to the ratio of the area under the plasma concentration-time profile of the victim drug in the presence and absence of a perpetrator (*AUC^i^/AUC*), which can be calculated by Equation (4) based upon the hepatic “well-stirred” model [58]. Equation (4) assumes no transient plasma binding (*f_μ_,_b_*) displacement exists, the fraction absorbed (*F_a_*) and the hepatic blood flow (*Q_H_*) are not modified by the presence of a perpetrator, and the concentration of the perpetrator does not change over time. Furthermore, the metabolism of the victim drug is assumed to only occur in the liver, and metabolism is the only route of elimination. That is, the total clearance (*CL*) equals the hepatic clearance (*CL_h_*). In this equation, *F_h_* represents the hepatic drug availability, *CL_h_* represents the hepatic clearance, *CL_μ__,int_* represents the unbound hepatic intrinsic clearance for a victim drug. *F_h_^i^*, *CL_h_^i^*, and *CL*^i^_μ_*_,int_* are the corresponding parameters in the presence of the perpetrator.

(4) $$\frac{AUC_{p.o.}^{i}}{AUC_{p.o.}}=\frac{F_{h}^{i}}{F_{h}}\times \frac{CL_{h}}{CL_{h}^{i}}=\frac{\frac{Q_{H}}{Q_{H}+f_{\mu ,b}\times CL_{\mu ,int}^{i}}}{\frac{Q_{H}}{Q_{H}+f_{\mu ,b}\times CL_{\mu ,int}}}\times \frac{\frac{Q_{H}\times f_{\mu ,b}\times CL_{\mu ,int}}{Q_{H}+f_{\mu ,b}\times CL_{\mu ,int}}}{\frac{Q_{H}\times f_{\mu ,b}\times CL_{\mu ,int}^{i}}{Q_{H}+f_{\mu ,b}\times CL_{\mu ,int}^{i}}}=\frac{CL_{\mu ,int}}{CL_{\mu ,int}^{i}},$$

The determination of *CL_μ__,int_* requires the in vitro enzyme kinetic studies, which are based upon the classical Michaelis–Menten equation (Equation (5)). *v*, *V_max_*, *K_m_* and *S* are the observed rate of metabolism (i.e., *CL_μ__,int_*), the maximal velocity, the Michaelis–Menten constant, and the victim concentration, respectively. When the concentration of a substrate is much lower than its apparent *K_m_*, the *CL_μ__,int_* equals to *V_max_*/*K_m_* [6].

(5) $$v=\frac{V_{max}\times S}{K_{m}+S},$$

In the presence of a perpetrator (inhibitor or inducer), the victim values of *V_max_* and *K_m_* will change. Accordingly, the ratio of *CL_μ__,int_/CL^i^_μ__,int_* can translate to the “R values”, such as *R_rev-c_*, *R_MBI_*, and *R_ind_*, which are, respectively representing the impact of competitive inhibition, MBI, and induction on *CL_μ__,int_*. The standard calculation of these “R values” follows the Equations (6)–(8) according to the newest FDA DDI guideline [28]. In these equations, *I_max,μ_* is the maximal unbound plasma concentration of perpetrator drug at steady state, *K_i,μ_* is the unbound inhibition constant, and *K_I,μ_* is the unbound inhibitor concentration causing half-maximal inactivation.

(6) $$R_{rev-c}=1+\frac{I_{max,\mu }}{K_{i}{}_{,\mu }},$$

(7) $$R_{MBI}=1+\frac{k_{inact}\times I_{max,\mu }}{k_{deg}\times (K_{I,\mu }+I_{max,\mu })},$$

(8) $$R_{ind}=1+\frac{E_{max}\times I_{max,\mu }}{EC_{50}+I_{max,\mu }},$$

Thus, the DDI potential (*AUC^i^/AUC*) can be expressed directly by in vitro enzyme kinetic parameters after substituting Equations (6)–(8) into Equation (4). Taking competitive inhibition as an example, the extent of DDIs could be displayed by Equation (9), which represents the basic model of DDIs.

(9) $$\frac{AUC_{p.o.}^{i}}{AUC_{p.o.}}=\frac{CL_{\mu ,int}}{CL_{\mu ,int}^{i}}=1+\frac{I_{max,\mu }}{K_{i,}{}_{\mu }},$$

The basic models based on these “R values” are the most common methods adopted in DDI evaluation. For CYPs-mediated DDIs, if *R_rev-c_* ≥ 1.02, *R_MBI_* ≥ 1.25, or *R_ind_* ≤ 0.8 [59], the FDA suggests conducting further investigation of the DDI potential in mechanistic models or clinical DDI study with a sensitive probe substrate [28]. For transporter-mediated DDIs, the criteria differ [28]. If *I_gut_*/*IC_50_* or *K_i_* ≥ 10 (*I_gut_* = dose of inhibitor/250 mL), the investigational drug (administered orally) has the potential to inhibit *P-gp* or BCRP in vivo. If 1 + ((*f_u,p_* × *I_in,max_*)/*IC_50_*) ≥ 1.1, (where *I_in,max_* is the estimated maximum plasma inhibitor concentration at the inlet to the liver), the investigational drug has the potential to inhibit OATP1B1/3 in vivo. Moreover, if *I_max,u_*/*IC_50_* value is ≥ 0.1, the investigational drug has the potential to inhibit OATs, OCTs, MATEs in vivo.

### 4.2. Mechanistic Static Model

The aforementioned equations are all based on the assumption that metabolism in the liver is the only route of the victim’s elimination, which is obviously hard to match the real situation. Considering the oral administration, the metabolism often involves other organs (e.g., gut and kidney) with different enzymes or transporters located. To account for this discrepancy, the “Modified Rowland–Matin model” (Equation (10)) was proposed, where *f^i^* means *f_m_* or *f_t_* as mentioned earlier [60,61,62]. The utility of this model is apparent when the victim is eliminated by several routes or involved multiple enzymes/transporters, as the percent contribution to the total clearance can be factored, and only the affected fraction (*f^i^*) is inhibited.

(10) $$\frac{AUC_{p.o.}^{i}}{AUC_{p.o.}}=\frac{CL_{\mu ,int}}{CL_{\mu ,int}^{i}}=\frac{1}{\frac{f^{i}}{1+\frac{I_{\mu }}{K_{i}}}+(1-f^{i})},$$

A limitation of the “Modified Rowland–Matin model” is the inability to distinguish the relative contributions of the enzyme- and transporter-mediated DDIs. For example, when an enzyme inhibitor is a substrate to a hepatic uptake transporter, the unbound intracellular concentration available at the target enzyme is different from the unbound plasma concentration [63], and the permeability of drug through membranes becomes a rate-controlling process for hepatic clearance. In this case, the “Extended clearance concept” (Equation (11)) can clarify the primary role of transporters and incorporate the co-contribution of the enzymes and transporters to intrinsic hepatic clearance [56,64].

(11) $$CL_{\mu ,int,h}=(SF\times PS_{uptake}+PS_{dif,inf})\times \frac{CL_{\mu ,int,met}+PS_{efflux}}{PS_{dif,eff}+CL_{\mu ,}{}_{int,met}+PS_{efflux}},$$

In this equation, *PS_uptake_* represents the basolateral active uptake clearance, which can be obtained from in vitro uptake experiments utilizing hepatocyte or recombinant cell line systems, and *PS_dif,inf_* represents the basolateral passive diffusion. *PS_efflux_* is intrinsic biliary clearance mediated by apical efflux transporters, derived from sandwich culture or vesicle experiments. *CL_μ,int,met_* represents the total intrinsic metabolic clearance, and it is determined by conventional in vitro incubation systems (e.g., microsomes) [65,66,67]. *SF* represents the empirical scaling factor for matching the in vivo *CL_μ,int,h_* [68].

By integrating the “extended clearance concept” with the specific transporter and enzyme inhibition/induction interactions, the most informative mechanistic static model—“net effect model” is obtained [69]. For instance, the competitive inhibition can be described by Equation (12) using this kind of mechanistic model.

(12) $$CL_{\mu ,int,h}^{i}=(\frac{SF\times PS_{uptake}}{R_{uptake}}+PS_{dif,inf})\times \frac{\sum \frac{CL_{int,cyp}}{R_{cyp}}+\frac{PS_{efflux}}{R_{efflux}}}{PS_{dif,inf}+\sum \frac{CL_{int,cyp}}{R_{cyp}}+\frac{PS_{efflux}}{R_{efflux}}},$$

where *R_uptake_*, *R_cyp_* and *R_efflux_* represent the competitive inhibition terms for active hepatic uptake, particular CYP isoform and biliary efflux transport, respectively. This model has been validated for quantitative DDI predictions involving CYP enzymes and OATP1B by using a set of ten substrate drugs and five inhibitor drugs [66].

## 5. Dynamic PBPK Model for PK DDIs

### 5.1. Concept of PBPK Model

**Figure 2:**
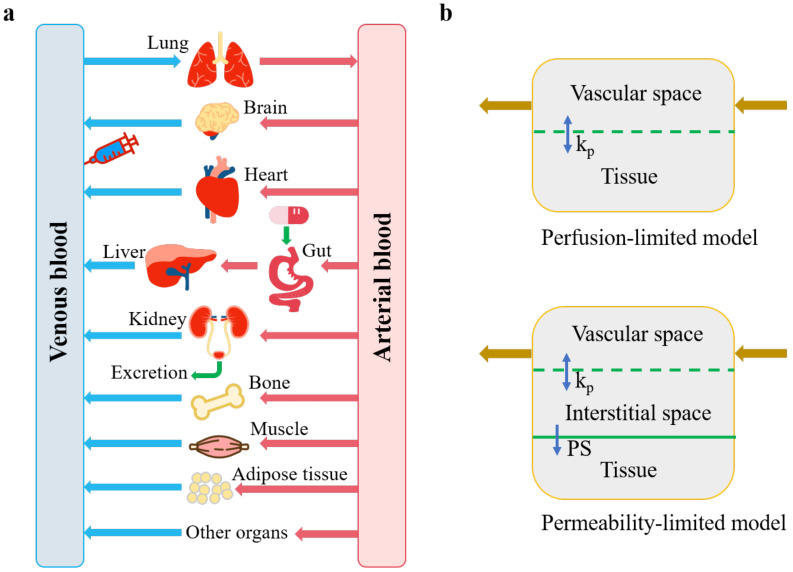
A generic physiologically based pharmacokinetic (PBPK) model (**a**) adapted from Shin, H.K. et al. [73], and 2 types of tissue model structure (**b**) adapted from Yuan, D. et al. [74]. *k_p_*, tissue partitioning coefficient, namely concentration ratio between tissue and blood at steady state; *PS*, membrane permeability coefficient.

The PBPK model consists of a series of linked differential equations that are solved numerically on the computer to track the time course of drug concentrations in blood and tissues after dosing. A whole-body PBPK model is a mathematical representation of the body that integrates the physiology data, the organs that are most relevant to the absorption, distribution, excretion, and metabolization of the drug, and blood connections between the organs (Figure 2a). These organs are typically lung, brain, heart, gut, liver, kidney, adipose tissue, bone, and muscle, and each one of them is characterized by a combination of associated blood-flow rate, tissue volume, tissue-partition coefficient (*K_p_*), and permeability [70,71]. Generally, PBPK models assume that each tissue compartment is well-stirred and perfusion rate limited, whereas permeability-limited distribution or disposition needs to be considered for large, hydrophilic molecules or transporter-dependent drugs (Figure 2b) [72].

The major PBPK applications (among PBPK-related publications) were associated with the study design, predicting formulation effects and metabolic DDIs, while studying the fate of drugs in special populations, predicting kinetics in early drug development, and investigating transporter-mediated DDIs have increased proportionally over the last decade [75]. In recent surveys of the PBPK-based submissions to FDA [76,77], about 56–67% of the submissions used PBPK modeling to evaluate the drug interaction potential.

### 5.2. Strategy of PBPK Modeling to Solve PK DDIs

**Figure 3:**
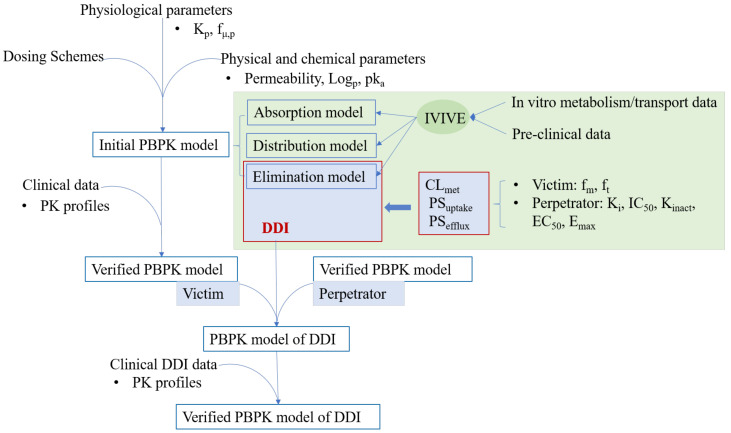
Workflow of PBPK modeling for the prediction of cytochromes P450 (CYP)/transporter-mediated DDIs.

The PK DDIs between victim and perpetrator drugs in vivo is a dynamic process, and PBPK modeling is a perfect approach to characterize this time-varying interaction, thus enables more accurate predictions of DDIs than static models. Besides, PBPK models are embedded with the variance of extrinsic and intrinsic factors (e.g., CYPs or transporter abundance and activity). The simulation and prediction results in the virtual population can not only indicate the case of the average population but also account for the case of extreme individuals [78]. The use of PBPK modeling to evaluate DDI risk is usually carried out in a stepwise manner. First, separate PBPK models for the victim and perpetrator drugs should be developed and verified. Briefly, the physiochemistry information (e.g., *Log P* and *pK_a_*) and in vitro metabolism/transporter data of the victim/perpetrator are plugged directly into a PBPK software tool, together with the integrated physiological parameters in the software, to obtain the preliminary parametrized initial model for the victim or perpetrator. The initial models are then verified/refined with clinical data (e.g., the PK profiles after intravenous or oral administration). Second, these two verified PBPK models (victim PBPK model and perpetrator PBPK model) are linked together by specific equations informed by the DDI mechanism derived from in vitro studies. Third, the DDI potential is predicted and verified with observed in vivo data (e.g., PK profiles, *AUC* and *C_max_* ratios). The workflow for using PBPK modeling to predict CYPs/transporter-mediated DDIs are described in Figure 3 [38]. Notably, the victim PBPK model should integrate delicate system data (e.g., enzyme and transporter expression levels) and drug-related information (e.g., enzyme-level based metabolism and transport-based uptake/efflux rates) [79,80]. Also, it is necessary for the victim PBPK model to ensure both the plasma concentration versus time profile and the fraction of the systemic clearance via the enzyme/transporter in question are correctly captured [81]. For the perpetrator PBPK model, it is crucial to correctly predict the unbound concentrations at the site of the drug interaction (e.g., intestine, liver, or kidneys) and provide accurate estimates of the interaction parameters (e.g., *K_i_*, *E_max_*). The above aspects are of key importance to simultaneously evaluate multiple inhibitions and induction mechanisms and to accurately predict the potential of DDIs [81,82].

The approaches to building a PBPK model have been well discussed in detail by Shebley et al. [81]. Usually, the bottom-up approach is the preferred option because it provides the mechanistic understanding of the drug’s metabolism and excretion based on in vitro data of the CYPs and transporters of interest. When the in vitro data are not available, the retrograde model based on the top-down or middle-out approaches can be applied. The frequently used PBPK software tools that possess capabilities for DDIs modeling are PK-Sim [83], Simcyp [84], and GastroPlus™ [85].

### 5.3. Key Differential Equations for DDIs in PBPK Modeling

**Figure 4:**
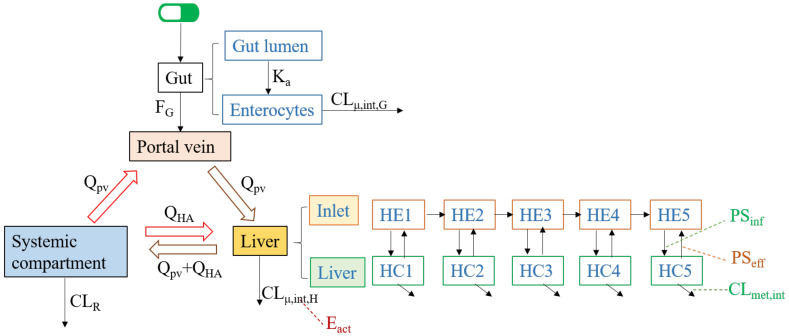
Sketch for major compartmental modules and related parameters. The subscripts of *pv, G*, *H*, *HA* and *sys* represent the portal vein, gut, liver, hepatic artery and systemic compartment, respectively. *Q* is the blood flow rate, *K_a_* is the absorption rate constant, *F_G_* is gut availability after intestinal metabolism; *CL_μ,int,H_* and *CL_μ,int,H_* are the unbound intrinsic clearance in liver and enterocytes. *PS_inf_* is the overall influx intrinsic clearance, *PS_eff_* is the overall efflux intrinsic clearance, and *CL_met,int_* represents the hepatic metabolism by CYPs.

Depending on the critical elimination pathways of a victim drug, the CYPs and transporters may coordinately contribute to the drug’s disposition. The CYPs mainly participate in hepatic and intestinal metabolism, the *P-gp* and BCRP transporters usually involve in the PK DDIs in the processes of intestinal absorption, bile secretion (canaliculus efflux) and renal secretion and the OATP transporters may dominate the hepatic clearance [20,25,86]. A clinically relevant DDI may occur when the perpetrator affects one of the main pathways of the victim drug. To give an in-depth understanding of how various interactions between victim and perpetrator are handled by the PBPK modeling approach, here we summarized the key differential equations for different DDI mechanisms. Considering the in vivo main pathways of a drug, the key differential equations for the DDIs within a simplified PBPK model concentrate on three modules—Portal vein (where the drug firstly comes into the systemic circulation from the gut), Liver (where the drug is metabolized or biliary excreted) and Systemic compartment (containing blood vessels, kidneys, and other non-eliminating organs). The simplified structure for these three modules is shown in Figure 4 [20,87]. Renal drug clearance (*CL_R_*) is defined by the sum of glomerular filtration (*CL_fil_*) and tubular secretion (*CL_sec_*) minus tubular reabsorption (*CL_reabs_*). Most renal DDIs are linked to inhibition of drug transporters (OATs, OCTs) for tubular secretion. The static or dynamic models to evaluate the transporter-mediated DDIs in the kidney follow the same principles as the transporter-mediated DDIs in the liver/intestine [88,89]. For simplicity, the transporter-mediated DDI in renal elimination is not presented below.

When the intestinal or hepatic metabolism is the major elimination pathway and the drug entering the liver is a perfusion-limited process, the concentrations of the victim drug in the portal vein, liver, and systemic compartment can be described by Equations (13)–(15) [87].

(13) $$\frac{{dC}_{pv}}{dt}\times V_{pv}=\left(C_{sys}-C_{pv}\right)\times Q_{pv}+f_{a}\times \frac{Q_{G}}{Q_{G}+f_{\mu ,G}\times CL_{\mu ,int,G}(t)}\times k_{a}\times D\times e^{-k_{a}\times t},$$

(14) $$\frac{{dC}_{liver}}{dt}\times V_{liver}=Q_{pv}\times C_{pv}+Q_{HA}\times C_{sys}-[f_{\mu ,B}\times CL_{\mu ,}{}_{int,H}(t)+Q_{pv}+Q_{HA}]\times C_{liver},$$

(15) $$\frac{dC_{sys}}{dt}\times V_{sys}=(Q_{pv}+Q_{HA})\times C_{liver}-(\frac{CL_{R}}{f_{\mu ,B}}+Q_{pv}+Q_{HA})\times C_{sys},$$

where *C* is the unbound drug concentration, *V* is the compartmental volume, *f_a_* means the fraction of dose absorbed from the gut lumen, *f_μ,G_* and *f_μ,B_* represents the unbound drug fraction in gut and blood, and *D* is the total drug amount.

If the hepatic uptake transporters such as OATP1B1/3 and OATP2B1 dominate the drug elimination, the drug entering the liver becomes a permeability-limited process. In this case, the liver can be subdivided into the extrahepatic (inlet) and hepatocellular (liver) compartments, which were divided into five small compartments sequentially connected by hepatic blood flow to mimic the dispersion model [90]. The mass balance differential equations for these two compartments could be described by Equations (16) and (17) [20]:

(16) $$\frac{{dC}_{inlet}}{dt}\times V_{inlet}=Q_{h}(C_{\mathrm{sys}}-C_{inlet})-f_{\mu ,B}\times PS_{inf}\times C_{inlet}+f_{\mu ,H}\times PS_{eff}\times C_{liver},$$

(17) $$\frac{dC_{liver}}{dt}\times V_{liver}=f_{\mu ,B}\times PS_{inf}\times C_{inlet}-f_{\mu ,H}\times (PS_{eff}+CL_{met,int})\times C_{liver},$$

where *Q_h_* is the hepatic blood flow rate, *PS_inf_* consists of the active uptake intrinsic clearance on the basolateral membrane by uptake transporters (*PS_uptake_*) and the influx intrinsic clearance by passive diffusion on the basolateral membrane (*PS_dif,inf_*), and *PS_eff_* consists of the intrinsic efflux clearance of canaliculus efflux transporters (*PS_efflux_*) and the intrinsic efflux clearance by passive diffusion on the basolateral membrane (*PS_dif,eff_*).

A perpetrator drug affects the victim drug by increasing or decreasing the victim’s *CL_μ,int_*, *PS_uptake_* or *PS_efflux_*. For enzyme-based metabolism, Equation (18) shows the *CL_μ,int_* of the victim drug in the absence of the perpetrator, and Equation (19) indicates the dynamic changes of the *CL_μ,int_* of the victim drug in the presence of the perpetrator. Regarding the transporter-mediated elimination, the impact of the perpetrator on the *PS_uptake_* and/or *PS_efflux_* of the victim drug can be determined in a similar way as *CL_μ,int_* [82]. If intestine clearance can be ignored and *CL_μ,int,h_* in the liver is known, *CL_μ,int_*, *PS_uptake_*, *and PS_efflux_* can be obtained from Equation (11) (“extended clearance concept”) via back-calculation.

(18) $${CL}_{\mu ,int}=\frac{V_{max}\times SF}{K_{m}+f_{\mu ,B}\times C_{liver/gut}},$$

(19) $${CL}_{\mu ,int}(t)=\frac{{CL}_{\mu ,int}}{R_{rev-c}}\times \left[1+f^{i}{}_{cyp}\times \left(\frac{E_{act}(t)}{E_{0}}-1\right)\right],$$

where *f^i^_cyp_* is the fractional CYP-mediated metabolism to *CL_μ,int_*, *E_act,H_* is the active amounts of the enzyme in the liver, and *E_0_* represents the enzyme amounts at baseline. *R_rev-c_* indicates the competitive inhibition.

As *CL_μ,int_* is related to the amount and activity of the metabolizing enzyme (*E_act_*), the *CL_μ,int_* would change along with the *E_act_* if the perpetrator inactivates or induces the metabolizing enzyme. This time-varying amount of the active enzyme can be described by Equation (20). *R_ind_* and *R_MBI_* indicate the induction and MBI by the perpetrator.

(20) $$\frac{dE_{act}{}_{,H}}{dt}=-k_{deg}\times E_{0}\times R_{ind}-E_{act}{}_{,H}\times R_{MBI},$$

To sum up, a PBPK model of DDI is a mathematical model constructed by the above key equations. The Equations (13)–(17) are differential expression indicating the flow of drug along with blood in the body, and the Equations (18)–(20) account for the drug interaction through a dynamic change of *CL_int_* and *E_act_*.

## 6. Current Status and Future Perspectives

The static model assumes that the concentration of a perpetrator is at the maximum concentration and tends to overpredict DDI risk. Different from the static model, the PBPK approach considers the temporal changes in inhibitor and substrate concentrations, the complex dosing regimens of the inhibitor and substrate, and the time-dependent changes in enzyme abundance, which is especially important for MBI and induction. Therefore, the PBPK model allows a full characterization of the in vivo concentration-time course and a more accurate determination of the magnitude of DDIs. In addition, the PBPK modeling allows high confidence in prospective DDI predictions considering the interindividual variability in enzyme expression levels arising from genetic, anatomical, demographic, and pathophysiological differences. Overall, PBPK models are useful tools to aid the design and interpretation of clinical drug-interaction studies and are of great value for assessing and delineating DDIs potential in patient populations.

Yet, as an advanced approach, the PBPK modeling relies on the quality of in vitro data and the accuracy of IVIVE [79]. Moreover, compared to CYP enzymes, the experience of using the PBPK model for transporter-based DDIs is limited, and the corresponding predictive performance is not that well. This is largely due to knowledge gaps in transporter biology and limited experience in determining and modeling the kinetics of transporters. The poor IVIVE for transport-related clearance and DDI prediction may be addressed by ensuring that each in vitro system responds appropriately to test substrates and inhibitors. One direction in this field is to screen the specific endogenous biomarkers for drug transporters, and this may aid the DDI risk assessment. There have been some potentially useful biomarkers reported for transporter functions, and they were recently reviewed [91]. For instance, the biomarkers for OATP1B hepatic uptake transporter includes coproporphyrins I and III (CP-I/III), tetradecanedioate, and glycochenodeoxycholate sulfate, and the biomarker N1-methylnicotinamide is targeted for MATE-mediated renal secretion. On the other hand, the gap of accurate DDIs prediction in tissues is also needed to be filled. The use of positron emission tomography (PET) imaging technology may be a doable way to identify the rate-determining process in tissues by detecting the tissue time-course of the radiolabeled transporter substrates [92,93]. In the future, PBPK modeling of the transporters is expected to approach the state of maturity, like currently the case of CYPs, by using the well-established screening assays to generate reliable data used for IVIVE.

Another important question to answer is when to use the static or the dynamic model for the DDI evaluation. The US FDA has made a good recommendation based on the typical basic model, modified Rowland–Martin model and PBPK model. First, one can start with the basic model and compare the calculated “R values” to predefined cutoff criteria to determine whether it is possible to rule out the potential for a DDI [28]. Second, if the basic model does not rule out the potential for DDIs, additional modeling analyses, using modified Rowland–Martin model or PBPK models to proactively understand the DDI risk and avoid unnecessary DDI trials are suggested. Last, if the predicted *AUC^i^/AUC* of a sensitive index substrate in the presence and absence of an investigational drug is ≤0.8 (induction) or ≥1.25 (inhibition) based on mechanistic models, and in vivo DDIs study is suggested to verify/negate the model predictions [77].

In recent years, we have witnessed an acceleration of the growing-up of PBPK [94]. With the mature of in vitro technologies and the buildup of PBPK system knowledge (enzyme/transporter abundance, physiological parameters, the effect of disease, age, sex, and polymorphism status), the empirical static approaches may be phased out. To promote the use of PBPK modeling, investment in education is needed to train more specialists in modeling and simulation. In addition, knowledge-sharing should be advocated in this field. This can be done by submitting the PBPK substance models, system parameters, and DDIs cases to open and publicly available platforms/ repositories.

## 7. Conclusions

This review has elucidated the mechanisms, the in vitro methods, and the static or dynamic models for CYPs/transporter-mediated PK DDIs. A perpetrator drug could inhibit or induce the CYPs/transporters responsible for the elimination of the victim drug, resulting in an increase or decrease of the victim’s exposure in vivo. To evaluate the DDIs mechanism and magnitude, HLMs, hHEPs and recombinant cell systems could be employed to determine the perpetrator’s intensity of influence to the victim and the victim’s susceptibility to interaction. The classic static models such as the basic model, the modified Rowland–Matin model, and the net effect model could be used as startup approaches to assess the potential risk of DDIs. As a dynamic and most informative in silico DDI evaluation tool, the PBPK model is increasingly advocated for DDIs predictions, particularly in cases where static models are inadequate to capture the overall DDI liability. Currently, there is still a big challenge to fill the data availability gap of transporters to improve the quality of DDI predictions, while the ongoing work and efforts could definitely shape the future.
